# Straight Line Foraging in Yellow-Eyed Penguins: New Insights into Cascading Fisheries Effects and Orientation Capabilities of Marine Predators

**DOI:** 10.1371/journal.pone.0084381

**Published:** 2013-12-18

**Authors:** Thomas Mattern, Ursula Ellenberg, David M. Houston, Miles Lamare, Lloyd S. Davis, Yolanda van Heezik, Philip J. Seddon

**Affiliations:** 1 Department of Zoology, University of Otago, Dunedin, New Zealand; 2 Department of Conservation, Science and Capability, Auckland, New Zealand; 3 Department of Marine Science, University of Otago, Dunedin, New Zealand; University of Lethbridge, Canada

## Abstract

Free-ranging marine predators rarely search for prey along straight lines because dynamic ocean processes usually require complex search strategies. If linear movement patterns occur they are usually associated with travelling events or migratory behaviour. However, recent fine scale tracking of flying seabirds has revealed straight-line movements while birds followed fishing vessels. Unlike flying seabirds, penguins are not known to target and follow fishing vessels. Yet yellow-eyed penguins from New Zealand often exhibit directed movement patterns while searching for prey at the seafloor, a behaviour that seems to contradict common movement ecology theories. While deploying GPS dive loggers on yellow-eyed penguins from the Otago Peninsula we found that the birds frequently followed straight lines for several kilometres with little horizontal deviation. In several cases individuals swam up and down the same line, while some of the lines were followed by more than one individual. Using a remote operated vehicle (ROV) we found a highly visible furrow on the seafloor most likely caused by an otter board of a demersal fish trawl, which ran in a straight line exactly matching the trajectory of a recent line identified from penguin tracks. We noted high abundances of benthic scavengers associated with fisheries-related bottom disturbance. While our data demonstrate the acute way-finding capabilities of benthic foraging yellow-eyed penguins, they also highlight how hidden cascading effects of coastal fisheries may alter behaviour and potentially even population dynamics of marine predators, an often overlooked fact in the examination of fisheries’ impacts.

## Introduction

Free-ranging marine predators rarely search for prey along straight lines [1]. Productivity and associated prey assemblages are heterogeneously distributed, requiring complex search strategies for success [2,3]. Foraging patterns generally resemble “Lévi-walks” where episodes of frequent, seemingly random course changes are interspersed with linear reorientation movements [1,3]. However, when travelling to foraging locations straight line movements are more common as deviations from a heading will prolong travel times and energy expenditure [4]. In order to maintain a linear course, animals must utilise environmental features for orientation [5]. The earth’s magnetic field, olfaction and/or sun and star compass all may provide important cues, at least in long distance movements [4].

Movement patterns of marine predators that target fisheries operations can appear considerably less random. Some seabirds are known to adopt foraging strategies in which they seek out fishing vessels to feed on bait or discards [6]. Such vessels are easy to locate in the planar sea-scape and may “guide” birds along linear trajectories. For instance, albatross and gannets have been found to follow fishing vessels for extended periods during which their movement patterns match a vessel’s course, exhibiting periods of straight line movements [7,8]. That these patterns have so far been observed only in volant seabirds seems logical, since locating fishing vessels should be greatly facilitated by the extended field of vision while flying. Likewise, flight allows seabirds to reach and follow mobile targets [9]. 

Penguins are one of the most important groups of marine predators in the southern hemisphere [10]. Since penguins are flightless it seems logical that there are no published records of penguins following fishing vessels. Instead penguins’ at-sea movements generally follow the principles of “Lévi-walks” outlined above [3,11]. However, at least in the case of one species, the yellow-eyed penguin (*Megadyptes antipodes*), foraging appears to be determined by factors that reduce the randomness of movement paths. Foraging tracks of this species have been found to be very consistent, even congruous if multiple foraging trips of individuals are examined [12,13]. This foraging strategy is facilitated by primary benthic foraging behaviour where seafloor features serve for orientation, and distinct patches of increased biodiversity (e.g. biogenic reefs, horse mussel fields) represent predictable, permanent targets for repeated visits, even over longer time periods [13,14]. 

During an impromptu foraging study of yellow-eyed penguins in response to a disease outbreak, we observed remarkable straight line foraging patterns akin to those reported in flying seabirds following fishing vessels. This triggered a more comprehensive study of foraging behaviour using GPS dive loggers aiming to (a) determine the prevalence of straight line patterns in the foraging behaviour of yellow-eyed penguins, (b) examine which environmental features the penguins might be utilising to maintain accurate linear courses, and (c) address the question why the penguins would forage along such linear trajectories.

## Materials and Methods

### Ethics statement

This research was approved by the University of Otago Animal Ethics Committee (AEC 32/03) and complies with the current laws of New Zealand. Entry and Research permits required for the work on the endangered Yellow-eyed penguin were issued by the Department of Conservation.

### Species and study site

**Figure 1:**
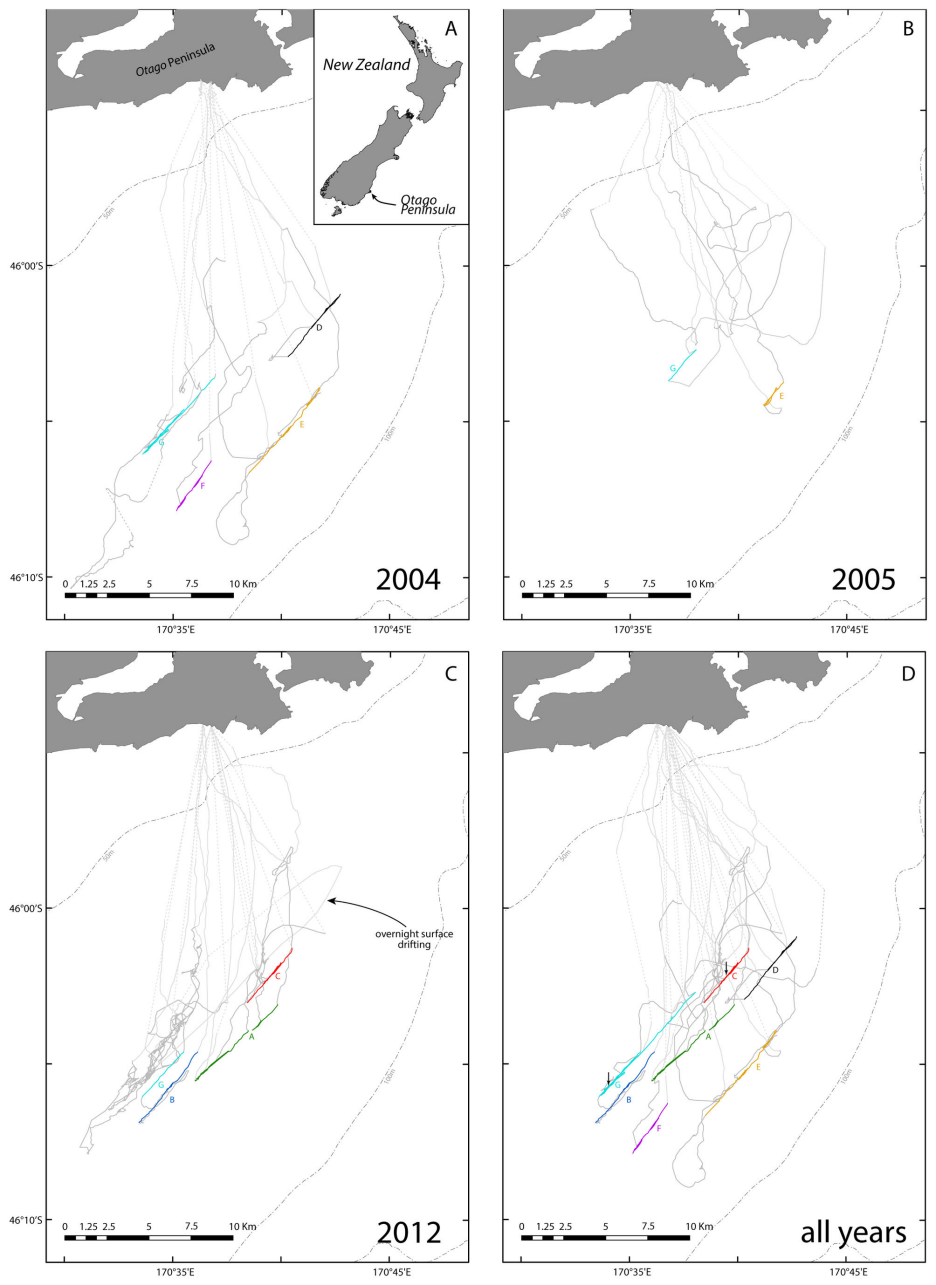
Foraging patterns of Yellow-eyed penguins. Mid-shelf foraging tracks of yellow-eyed penguins recorded in 2004 (A), 2005 (B) and 2012 (C) that feature straight-line patterns. Foraging track segments in light grey represent outgoing and incoming stages of foraging trips; dark grey segments highlight the foraging stage. Dashed line segments indicate where linearity of the track is a result of interpolation. Track portions that met line criteria (see Methods) are highlighted in different colours; line identifiers shown in capital letters of the same colour. Small arrows in (D) indicate sites of ROV deployments in February 2013. Trips with lines from all three seasons are combined in (D).

The yellow-eyed penguin is endemic to New Zealand and breeds on the sub-Antarctic Auckland and Campbell Islands, along the southeast coast of New Zealand’s South Island, and Stewart Island and its outliers. The species is classified Endangered in the 2013 Red list [15], and listed Nationally Vulnerable under the New Zealand Threat Classification System [16]. The world population is estimated to be around 1,700 breeding pairs, 60% of which are thought to occur in the sub-Antarctic region [17]. The Otago Peninsula (Figure 1) represents a mainland stronghold for the species and currently holds around 180 breeding pairs [17]. This study was conducted at the Boulder Beach complex (-45.897°S, 170.620°E), which represents the area with the highest density of yellow-eyed penguins on the Otago Peninsula. The area is subject to a long-term monitoring programme (since 1980s) and has received considerable research attention during the past two decades [18]. 

### Disease outbreak & foraging study

In late November and early December 2004, an outbreak of a disease later described as *diphtheritic stomatitis* affected most chicks along the Otago coast [19]. Unusually high mortality in young chicks triggered a series of immediate conservation actions including treatment with broad-spectrum antibiotics and supplementary feeding with glucose solution as well as this study. At the time, a potential connection between outbreak and prevailing sewage pollution along the inshore ranges of the Otago Peninsula was suspected. The present study was initiated to examine whether adult penguins from affected nests were visiting sea areas with increased pollution levels. On the basis of the observed foraging patterns the study was then expanded and further foraging studies occurred in December and January of the breeding seasons 2005 and 2012. *Diphtheritic stomatitis* did not occur in 2005. It was again present in 2012 but not to the extent of the 2004 outbreak.

### GPS dive loggers and deployment

The foraging behaviour of breeding yellow-eyed penguins was examined with GPS dive loggers (GPS TD log, earth&Ocean Technology, Kiel, Germany; dimensions: L100xW48xH24mm, mass: ca. 70g). The devices contain a GPS receiver to determine geographical position (error for most fixes <10 m [20]) and a depth sensor (resolution ~0.1 m) to record dive behaviour. All data are stored with an accurate timestamp. The devices were programmed to record depth data at 1 s intervals. The GPS receiver was pressure-activated and attempted to record a position after each dive (“upon resurfacing”). The device required on average 20 s to determine its position (“GPS fix”). If a bird remained at the surface <20 s, chances were that the device would not be able to get a GPS fix, a situation that primarily occurred at the beginning and the end of the foraging trips, i.e. when birds travelled to and from their main foraging areas [13]. Data could only be downloaded after device recovery. 

Devices were fitted to penguins’ lower backs with adhesive tape (Tesa® tape, No. 4651; Beiersdorf AG, Hamburg, Germany) [21]. Timing and duration of deployments differed between the three seasons. In 2004, the study concentrated on the first two weeks of December, i.e. the early chick rearing period [17]. A total of eight birds were fitted with GPS dive loggers. Deployment times were limited to two to three days per bird to allow quick re-deployment of the limited number of devices available. In the following season 2005, four birds that had exhibited linear foraging in 2004 were fitted with GPS loggers. As only a single GPS device was available, deployments occurred consecutively spanning the entire month December 2005 and the first week of January 2006. Devices remained on birds for up to six days. In 2012, logger deployments spanned the entire chick rearing period. A total of 11 penguins were fitted with GPS loggers between early December 2012 and late January 2013, with deployment times ranging between five and seven days. One of the birds (band number: 17395) had been part of the initial study in December 2004. 

### Data analysis

Yellow-eyed penguins do not show any marked sex differences in foraging behaviour and performance [12,13]; thus, we did not discriminate between sexes during data analysis. GPS dive logger data were analysed using custom-written software (T. Mattern, unpubl. data). GPS data were used to determine basic foraging parameters for each foraging trip, namely *travel distance* (determined from sum of linear distances between a foraging trip’s consecutive GPS fixes) and *foraging range* (maximum distance from nest site). Depth data served for the analyses of diving behaviour. Start and end times of dives were identified from timestamps of corresponding depth changes. By combining GPS and dive data it was possible to assign a geographical position to every dive. If a GPS fix was recorded during the surface interval immediately before a dive, we defined this position as the location of the dive. If the device failed to record a GPS fix before the dive, a linearly interpolated position was determined using timestamp and geographic position of last fix recorded before and first fix recorded after the respective dive. 

Dive events were accepted if depths >0.5 m were recorded and pressure changes lasted for 3 s or more. A range of dive parameters were calculated for each dive: *dive duration*, *bottom time* (i.e. time spent at depths >95% of maximum depth reached), *transit time* (i.e. time spent descending to and ascending from bottom phase depths), and *post-dive interval* (i.e. time spent at surface until onset of the following dive). Dives were categorized as either benthic or mid-water/travelling dives via dive profile analysis. Benthic dives were identified by comparing the measured maximum dive depth with the approximate water depth at the position where the dive occurred; water depths were determined from detailed nautical charts (BlueChart Pacific v9.5, Garmin MapSource). Additional criteria were applied, namely dive profiles with trapezoid shape and constant maximum depths during series of dives [13]. 

Yellow-eyed penguin foraging trips feature well-defined phases. An outgoing travel/search phase during which the birds tend to maintain a constant heading is followed by a foraging phase which is characterised by intensive diving behaviour and frequent course changes; an incoming travel phase during which birds perform shallow dives and cover large distances in short time completes a trip [13]. Trip phases were determined for all foraging trips. Assuming that frequent course changes indicate prey searching behaviour, we defined the end of the outgoing phase as the time and position after which a penguin changed its travel bearing at least three times by more than 45° within a 15 minute interval. Likewise, the onset of the incoming stage was defined as the position and time after which course changes >45° no longer occurred and the bird assumed a homeward bound bearing. 

GPS fixes recorded during the foraging phase of individual penguins were analysed in ArcGIS 10 (ESRI, Redlands, CA, USA). Straight line patterns during the foraging phase were visually identified from plots of foraging tracks where the birds maintained constant headings for at least 1,000 m. “Lines” were confirmed if they derived from GPS data of more than one bird, if an individual backtracked along the same line (i.e. turned around and swam in the opposite direction) or revisited the same line at a later stage of the foraging phase or on another foraging trip. To avoid interpolation bias, only data sequences were accepted where a GPS fix was obtained before each dive event. GPS fixes corresponding to the lines were isolated and data were converted to orthomorphic New Zealand Map Grid coordinates. A linear least square regression was fitted to line fixes of individual birds and regression residuals were used as measure of horizontal deviation; goodness of fit (r^2^) for all accepted lines was >0.99. For each line, *total line length* was calculated as the horizontal distance between the south-western and north-eastern extremes of all GPS fixes associated to the respective line. The penguins’ horizontal swimming speed was determined from time interval and distance between consecutive GPS fixes. Statistical analyses were carried out in R [22]. 

As supplementary materials we provide KMZ files that illustrate aspects for foraging patterns we observed throughout this study, namely three dimensional representations of selected foraging trips which combine GPS and dive data in pseudo-3D plots (see 13 for details), and 2D animations of movement patterns. The files are best explored in Google Earth [23] but can be imported in many other GIS or mapping software packages.

### Seafloor surveys

On 19 February 2013, we undertook a one-day cruise on the University of Otago Research Vessel Polaris II to the foraging grounds of yellow-eyed penguins from the Otago Peninsula. Two offshore stations were chosen that coincided with lines determined from penguin foraging tracks (Figure 1d). 

We surveyed the seafloor with a remote operated vehicle or ROV (LBV-150SE MiniROV (Seabotix San Diego, CA, USA) which transmitted live video footage from its internal camera (resolution: 720x576 pixels, 25 frames/s) via a 150 m tether to the vessel, where footage was recorded to a laptop computer. Additionally, a high definition camera (GoPro Hero3 Black Edition, Woodman Labs, USA) was attached to the top of the ROV which recorded high-definition, wide-angle video footage (resolution: 1920x1440 pixels, 48 frames/s) to internal memory that could only be accessed after recovery of the vehicle. The ROV featured two scaling lasers positioned 5 cm apart and measured depth and heading. A GPS dive logger with settings similar to the deployments on penguins was attached to the ROV to record entry and exit locations as well as depth profiles of the vehicle. 

## Results

### GPS tracking

GPS tracking of yellow-eyed penguins in all three years revealed distinctive straight-line foraging patterns (Figure 1a-c). In the breeding season 2004, 10 foraging trips were recorded from eight birds (Table 1). Seven trips went to the mid-shelf region located >15 km from the coast. On all of these offshore trips, the penguins foraged along a northeast-southwest axis. Five trips featured portions that met the straight line criteria. No straight line patterns were observed in three inshore trips performed by two birds.

**Table 1:** Foraging parameters of yellow-eyed penguins fitted with GPS dive loggers.

	**2004**	**2005**	**2012**
Number of Birds	8	4	11
Inshore trips (<15 km from coast)	3	12	33
Mid-shelf trips (>15 km from coast)	7	5	11
Trip duration (h)	15.5±4.1	9.1±2.6	9.9±4.6
Travel Distance (km)	54.5±12.0	30.5±10.3	33.6±18.9
Foraging Range (km)	21.1±5.9	11.0±3.1	10.8±6.2
Trips with lines	5	2	6
% trips with lines	45.5	11.8	25.0
Birds on lines	5	2	3
Duration foraging phase (h)*	7.4±2.8	6.4±0.8	5.3±2.6
Time on lines (h)	3.3±1.6	1.2±0.4	1.7±1.2

Values (derived from individual means when applicable) are given as mean±SD.

^*^ travelling phases at the beginning and end of each foraging trip are omitted, see methods for details.

The following year, GPS loggers were fitted to four of the penguins that had exhibited linear foraging patterns in the previous season. One of these birds never ranged further than 8 km from its nest site. The remaining three birds all performed a mix of inshore and mid-shelf trips, although the penguins overall foraged closer to the coast that year (Table 1). Two birds performed one trip each where brief segments met the straight-line criteria (Figure 1b). Neither of the two foraged along the lines they had visited before but both foraged along lines that were apparent in penguin tracks from the previous year. On the three remaining mid-shelf trips the penguins neither showed linear foraging patterns nor did show any affinity for northeast or southwest bearings that were apparent in 2004. 

In season 2012, 11 out of 44 recorded foraging trips went to the mid-shelf regions. Of these, six trips performed by three birds featured lines. One bird (band number 17935) revisited a specific line (“C”) on three different trips (Figure 1c, see also File S1 line-*C_3D-tracks.kmz*, Supporting Information). The same bird had also been equipped with a GPS logger in 2004 when it foraged along a different line (“E”, Figure 1a).

**Figure 2:**
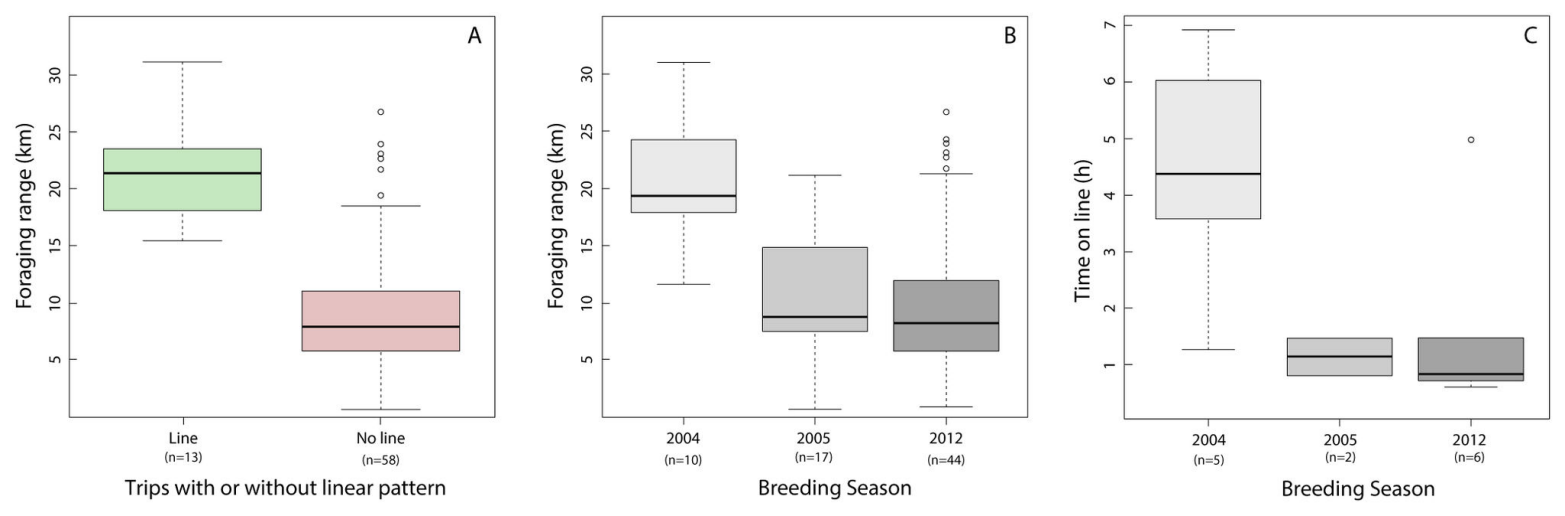
Comparison of basic foraging parameters in relation to breeding season and occurrence of linear patterns. Box-and-whiskers plots illustrate differences in foraging parameters between trips with and without linear patterns (A), and between the three breeding seasons (B & C). Bold horizontal lines indicate median and circles represent outliers. Note that graphs A and B are based on all recorded foraging trips, while for C only trips that met straight line criteria were used. Sample sizes are provided below x-axis labels.

The occurrence of linear foraging patterns was related to foraging ranges (Table 1, Figure 2a). While largely comparable between 2005 and 2012, foraging trips in 2004 were of longer duration (Kruskal-Wallis rank sum test: Χ^2^=7.74, df=2, p=0.02), the penguins travelled greater distances (Χ^2^=8.63, p=0.01), and ranged further offshore (Χ^2^=9.23, p<0.01, Figure 2b). Also, in 2004 nearly every second foraging trip featured lines, while in 2005 and 2012 linear foraging occurred considerably less frequently (Table 1). Similarly, penguins spent more time foraging on lines in 2004 when compared to the other two seasons (Χ^2^=9.67, p<0.01; Figure 2c). 

Overall, seven lines were discernible (Figure 1d). Four lines were apparent in the foraging tracks of single individuals; the remaining three lines were used by two to three birds (Table 2). Lines were approximately parallel to each other with an average south-west heading of 220±5°. Line lengths varied, with the longest line (“G”) spanning a total distance of 9.25 km. Some lines were well-defined with GPS fixes deviating by about 30 m from the lines’ trajectories over distances of several kilometres (Table 2). Five of the lines were utilised by penguins during single seasons, the remaining lines were apparent in 2004 and 2005 (“E”, Figure 1b&c), and in all three season (“G”, Figure 1a-c), respectively. On lines, 87.5-100% of the dives went to the seafloor where water depths ranged between 60 and 80 m (see also File S1 line-*C_3D-tracks.kmz*, Supporting Information).

**Table 2:** Geographical characteristics, foraging and diving parameters for eight lines determined from Yellow-eyed penguin foraging tracks recorded between 2004 and 2012.

	**Line ID**						
	**A**	**B**	**C**	**D**	**E**	**F**	**G**
***Line parameters***							
total line length (km)	6.65	6.15	4.00	4.95	9.10	3.65	9.25
heading (°)	228	220	221	221	220	217	223
number of birds	2	1	1	1	3	1	3
number of visits	3	1	3	1	3	1	5
Year(s) observed	2012	2012	2012	2004	2004,2005	2004	2004,2005, 2012
Number of GPS fixes	57	62	40	34	168	66	76
***Foraging parameters (mean±sd)***							
Time on line (h)	1.0±0.5	3.6	1.0±0.3	2.7	2.7±1.0	4.4	2.2±1.7
Horizontal deviation (m)	51.8±40.5	132.0±104.5	30.9±25.9	31.8±26.5	104.5±85.0	33.7±28.8	50.3±38.7
Horizontal speed (km/h)	3.8±2.2	3.0±2.6	2.6±2.7	3.1±1.8	2.2±1.5	2.9±1.9	3.0±2.2
No of dives	22±10	39±34	24±10	48	86±6	89	38±36
% benthic dives	94±5.2	96.7±4.7	97.1±4.2	87.5	97.7±1.5	98.9	97.5±4.4
Max dive depth (m)	66.2±5.3	62.0±3.8	62.5±0.7	67.9	77.3±0.2	71.6	61.0±4.1

Each line was followed by the penguins in either direction, i.e. southwest or northeast; some birds swam along the same line in both directions. The accuracy with which the penguins followed the linear courses varied between the different lines (Table 2, horizontal deviation: Χ^2^=127.31, df=6, p<0.01). Horizontal deviation did not relate to total line length (Pearson correlation: r=0.47, p=0.28) or number of GPS fixes (r=0.54, p=0.21); as such variations were line-specific rather than data-related artefacts. The three lines that were followed with highest precision (lines “C”, “D”, and “F”; mean horizontal deviation <40 m) were utilised by single individuals. Particularly lines “C” and “D” are noteworthy, because in both cases the penguins managed to revisit and precisely re-track the line at later stages of their foraging trip (line “D”) and on different foraging trips (line “C”) (see File S2 *straight-line-trip-animations.kmz*, Supporting Information). 

### ROV seafloor surveys

The first ROV deployment occurred towards the southern extreme of line “G” (S46.08°, E 170.57°, Figure 1d) where linear track segments of 2004, 2005 and 2012 data had overlapped suggesting that the penguins most likely utilised a permanent feature for orientation. The seafloor was surveyed for 15:26 minutes at depths of 64.8 to 65.3m during which the ROV covered a linear distance of approximately 180m. The ROV overall followed a southern trajectory and represented a cross-section of line “G”. 

The seafloor on line “G” consisted primarily of coarse sediment littered with fragments of large bivalve shells (see http://vimeo.com/64485882). No obvious bottom features were apparent that offered an explanation as to how penguins managed to maintain an accurate straight line course. Occasionally, individual horse mussels (*Atrina zelandica*) protruded from the otherwise featureless seafloor. Yellow-eyed penguin prey species such as juvenile forms of benthic blue cod (*Parapercis colias*) and tarakihi (*Nemadactylus macropterus*) were frequently seen throughout the survey as were sub-adult forms of the squat lobster (*Munida gregaria*). A single opalfish (*Hemerocoetes monopterygius*) was also observed, another important yellow-eyed penguin prey species. Brittle stars (*Ophiopsammus maculata*) were abundant throughout the entire duration of the survey. 

The second ROV deployment coincided with the central segment of line “C” (S46.03°, E170.66°, Figure 1d) which had been utilised by a single penguin on three foraging trips in December 2012. The ROV’s bottom time amounted to 21:17 minutes at water depths of 67.2 to 67.5m. The vehicle travelled around 230 m; during a portion of which it drifted with the strong currents. The ROV’s travel path ran in a north-eastern direction matching the trajectory of line “C” (see File S1 line-*C_3D-tracks.kmz*, Supporting Information).

The seafloor at this station was characterised by of a mix of sandy sediments that was also littered with shell fragments (see http://vimeo.com/64689982). With the exception of a small cluster of sponges and anemones that were encountered shortly after the ROV had reached the seafloor, there was little to no epibenthos. Blue cod again were abundant, but remained the only fish species that was encountered throughout the deployment. Just as at the first station, brittle stars dominated the benthos.

**Figure 3:**
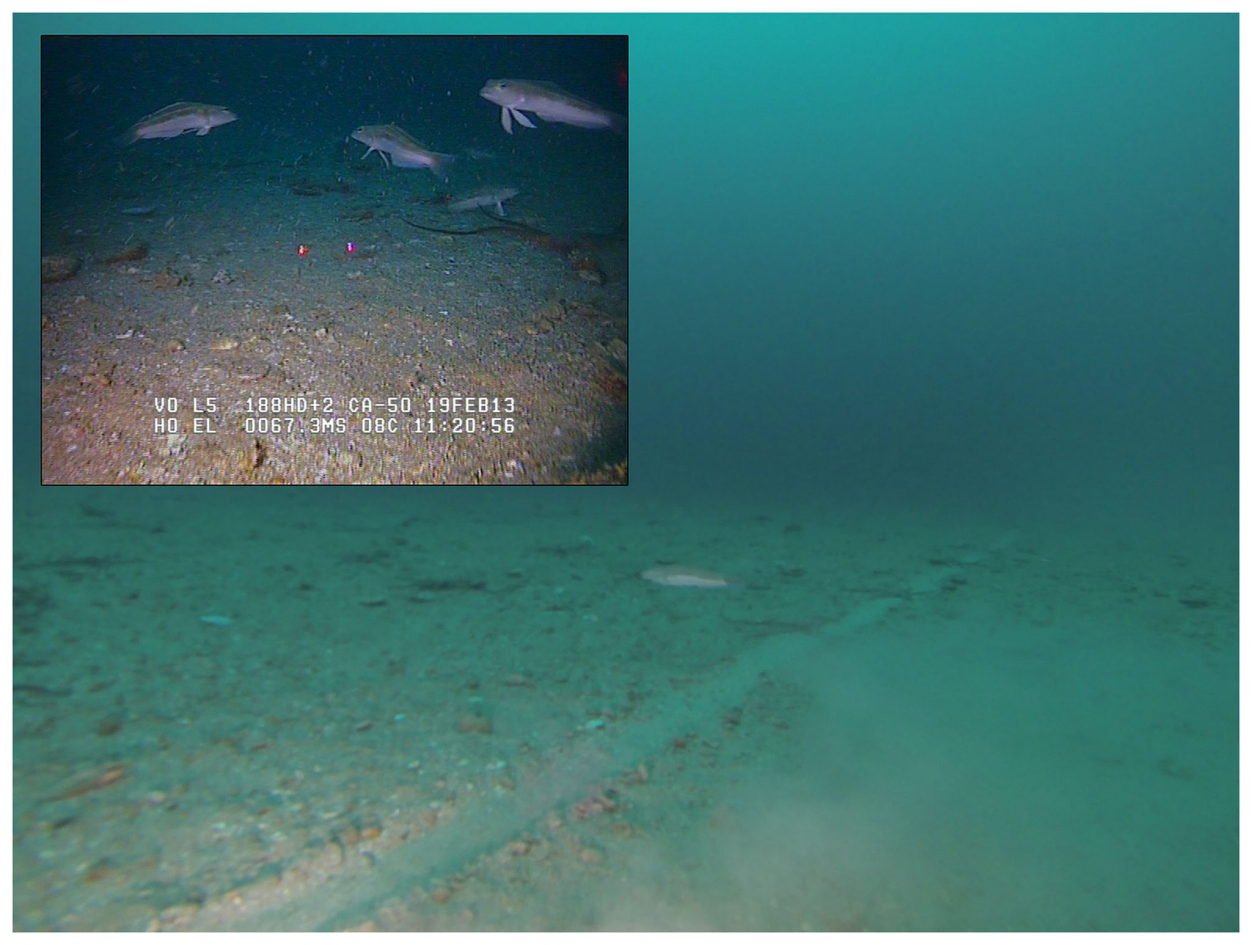
Screen capture of ROV footage recorded on line “C”. Main: Highly visible furrow running in a straight line along the seafloor at water depth of ca. 67 m. Note the echinoderms that have settled inside the furrow. Inset: Detail of blue cod in pursuit of ROV; scaling lasers represent 5 cm. See also (http://vimeo.com/64689982).

Ten minutes into the survey a linear furrow about 10-15 cm wide and 2 cm deep came into view (Figure 3). The mark ran in a straight line and was highly visible due to lighter coloured sediment inside the furrow. Due to the strong currents, manoeuvrability of the ROV was temporarily lost. The vehicle drifted with the current in a parallel trajectory to the furrow. Overall the furrow was in view for 4:43 minutes during which the ROV was constantly in motion. Judging from total deployment time and travel distance the ROV likely drifted along the furrow for a distance of at least 40 m, probably more. The furrow seemed to be undergoing slow erosion as some segments featured a visible berm, while the rims of other segments appeared bevelled or flat. Echinoderms had settled inside the furrow indicating that the mark was not a result of recent activities. Review of the high-definition footage seemed to briefly show a second scour mark in the distance, but this could not be confirmed. While drifting, the ROV repeatedly touched the bottom stirring up sediment. These sediment clouds seemed to be attractive to blue cod with individual fish actively pursuing the vehicle, apparently picking items from the stirred-up sediment.

## Discussion

### Prevalence of straight line foraging patterns

Yellow-eyed penguins from the Otago Peninsula regularly show straight line foraging behaviour. While at first it appeared to be an extraordinary occurrence in a “bad season” [24], the re-emergence of straight line patterns in subsequent seasons showed that this foraging behaviour happens on a regular basis, albeit with varying frequencies. The most important prerequisite for linear patterns is that the penguins forage further than 15 km from the coast. 

Foraging ranges in yellow-eyed penguins are a function of individual preferences and feeding conditions. A three year radio tracking study of penguins from the Otago Peninsula found that the birds have distinct individual centres of activity, that in some birds were closer to the shore while in others were located in the mid-shelf regions [12,25]. The same study also found that in years of poor breeding success, which is generally attributed to poor feeding conditions [26,27], yellow-eyed penguins were more likely to travel to the mid-shelf regions where linear foraging occurs. Our data reflected these patterns. 

In 2004, which demonstrably was a year of poor breeding success [19], most of the penguins foraged in the mid-shelf region and subsequently foraged along lines. However, only two of four birds that had foraged offshore did so in the following year when breeding success indicated better foraging conditions [28]. The penguin that was fitted with GPS loggers in 2004 and again in 2012 showed a preference for offshore foraging – and showed linear patterns on both occasions. Thus linear foraging patterns may occur more frequently in individuals that habitually forage in the mid-shelf region.

Linear foraging might have been occurring as early as the 1990s. Offshore foraging locations of yellow-eyed penguins determined during the radio tracking study between 1991 and 1993 were clearly distributed along the same northeast-southwest axis that we observed (see Figure 4 in [12]); some point sequences seem to be aligned along straight lines. The limited accuracy of position fixes determined via radio telemetry likely masks actual linear foraging patterns [29]. 

The apparent prevalence of linear foraging in yellow-eyed penguins indicates that this is a viable foraging strategy for the penguins, especially in years of poor food supply. Considering that linear foraging in seabirds has been attributed to interactions with fisheries operations the question arises whether this may also apply to yellow-eyed penguins. However, unlike flying seabirds that actively pursue fishing vessels to feed on discards [7,8], the backtracking and re-visitation of lines on different trips seems to rule out that the penguins were following boats. 

### Orientation cues in the marine environment

The orientational precision with which yellow-eyed penguins maintained straight line courses can only be explained through the use of clear environmental cues [5]. Despite its featureless appearance, the oceanic environment may offer plenty of navigational cues. Seabirds are believed to utilise the Earth’s magnetic field as well as olfactory cues for orientation and navigation [30,31]. However, it seems that both magnetic and olfactory cues operate principally on larger scales and are of greater importance for migratory species on long distance trips [4,32]. Temperature gradients and currents are believed to be part of a spatial reference frame utilized by marine animals for linear at-sea movement [33,34]. The Southland current moves towards the northeast along the Otago coast [35], and its flow direction approximates the trajectories of the lines, as an overnight surface drift pattern of one penguin demonstrates (Figure 1c). Swimming against or with the current might explain how penguins can stay on course while travelling along a line. However, it seems unlikely that an ocean current which is influenced by a multitude of dynamic physical processes (e.g. tides, storm events, temperature, salinity [36]) could provide spatially stable cues for orientation that allow penguins to repeatedly swim up and down exactly the same line, or relocate the same line on different foraging trips. Permanent topographical features offer a more viable explanation [4]. 

The yellow-eyed penguin is known to be a primarily benthic forager [12,13,27]. This diving strategy allows the birds to utilise visual cues on the seafloor and it has been suggested that the sedentary, non-migratory nature of the species would allow for the development of memorized landscape maps for navigation similar to homing pigeons [13]. Pigeons are known to utilise human infrastructure such as motorways and railways for way-finding [37]. 

Our survey of line “G” did not reveal any obvious bottom features that explained how three different penguins managed to swim along the same line over a timespan of nearly 10 years. However, straightness and length of the line suggest that environmental cues used by the birds must be well defined with little lateral variability. In this light, surveying the seafloor in a cross section of line “G” probably reduced the chance to detect such a feature. Another possible explanation for the absence of visual cues could be that they were of a non-permanent nature.

Indeed, our findings on the seafloor at the location of line “C” indicate that man-made cues prone to erosion over time provide yellow-eyed penguins the means for accurate way-finding.

### Fisheries-related seafloor disturbance as cues for orientation

As mentioned before, flying seabirds have been reported to actively pursue fishing vessels along straight line trajectories [7,8]. That the penguins used a similar strategy seems unlikely, since most penguins turned around to backtrack along the exact same line at least once. Likewise, it is hard to imagine that fishing vessels would fish along the exact same course on consecutive days which would be necessary to explain the movements of the penguin following line “C”. That the penguins were feeding on discards thrown overboard from the fishing vessel seems also unlikely in the light of the almost exclusive benthic foraging behaviour we observed (Table 2). The final point that speaks against penguins actively following fishing vessels are the highly variable horizontal swimming speeds as expressed by great standard deviations between consecutive fixes (Table 2); if the penguins were in pursuit of a boat, a more constant swimming speed could be expected. Instead, an indirect interaction between fishing activities and penguin foraging seems more plausible.

Bottom fishing gear such as trawls and dredges leave visible marks on the seafloor that may persists for weeks or even years [38]. The straight line furrow we encountered on line “C” most likely derived from an otter board of an inshore bottom trawl. Otter boards keep the mouth of the trawl net open and dig into the seabed creating sediment clouds that herd fish into the net [38]. The width and depth of the furrows created by otter boards depend on their size and weight. Heavy otter boards (2,300 Kg) create furrows with a width of 20 cm and depth of 10 cm [39]. In this case, the furrow was about half as wide, suggesting a smaller bottom trawl. Inshore fisheries targeting demersal tarakihi, gurnard (*Chelidonichthys kumu*) and red cod (*Pseudophycis bacchus*) use fishing gear with small otter boards (<500 Kg) [40].

In New Zealand, only vessels exceeding 28 metres in overall length are required to report their position at regular intervals via the Vessel Monitoring System (New Zealand Fisheries Act 1983). For the smaller inshore vessels like those operating in the mid-shelf regions off the Otago Peninsula only commercial fishing effort and commercial catch per management area are reported [41]; no detailed vessel tracks are available that would have allowed us to match the observed lines with actual vessel movements. However, bottom trawl activity (vessels <28m) reported for the area (SA 024) in which penguins forage was 55 vessel days in December 2012 and averaged 1,100 vessel days per year in the past decade [41]. This indicates frequent inshore fisheries activity within the penguins’ foraging range. Hence, line encounters by penguins may be a common occurrence. 

The furrow’s high visibility (Figure 3) makes it easy to locate and renders it an obvious orientation aid. Location and trajectory of the furrow (as determined from ROV deployment and recovery locations) match exactly that of line “C”, which is unlikely a coincidence. As line “C” was observed in December 2012 we can assume that the furrow was at least 10 weeks old when we discovered it. Otter board tracks can remain readily observable after such a time period [38,42]. This underlines the persistence of such markings and their long-term availability as orientation features. But what could motivate penguins to swim along furrows?

### Predictable prey assemblages over disturbed seafloor

The physical impact of demersal fishing gear on species living on or in the seafloor can be substantial [43]. Exposed, damaged or moribund animals (e.g. bivalves, echinoderms, crustaceans) are often left in the wake of bottom trawls, which in turn attract benthic scavengers [44]. Blue cod, an important prey for yellow-eyed penguins [27], falls into this category [45]. Blue cod were abundant during the ROV deployments, displaying inquisitive behaviour by actively following the vehicle, occasionally catching food items from stirred up sediment, presumably small crustaceans (i.e. isopods, amphipods) which usually dominate the diet of blue cod of the observed size [45,46] (Figure 3). Large-scale disturbance by bottom fishing is probably similarly attractive, if not more so. The abundance of scavengers should be greatest shortly after the occurrence of the trawl and then diminish once exposed or damaged prey has been consumed. The presence of blue cod during the ROV deployment at line “C” seems to suggest that fished areas remain attractive to scavengers for extended periods after the actual fishing event. While frequent bottom trawling is known to reduce habitat complexity and benthic biodiversity, it can simultaneously lead to a long term increase of opportunistic invertebrate species that in turn are important prey for commercial fish [44,47]. The abundance of blue cod we observed suggests that similar situation might apply in the mid-shelf region off the Otago Peninsula.

This could render foraging even along older trawl marks a viable foraging strategy for the penguins, especially in years when long foraging ranges indicate that food supply closer inshore is suboptimal [12]. As such, linear foraging may represent a fall-back strategy and could be more frequent in years of reduced breeding success. The fact that blue cod dominates the diet of yellow-eyed penguins in such years certainly supports this idea [14,27,48].

### Reduced diet quality when foraging along lines?

While it has been suggested that yellow-eyed penguins might be selective foragers which actively pursue the most nutritious prey [48], subsequent research indicates that diet composition is more likely related to prey availability in different foraging zones [27]. Yellow-eyed penguins breeding along the northeast coast of Stewart Island, some 200 km south of the Otago Peninsula have been found to feed almost exclusively on a diet of blue cod [14]. The home ranges of these birds are subject to extensive demersal oyster fisheries [18] which diminishes habitat complexity and species diversity of the benthic ecosystem [49]. Blue cod’s tolerance to benthic disturbance [50] is likely to make them the most abundant species available for the penguins. However, a monotonous diet of blue cod may come at the expense of reduced chick survival. The reproductive output of yellow-eyed penguins in the vicinity of oyster operations is very low and years of total breeding failure have been reported [51]. The nutritional value of a blue cod diet is low and fish caught by the penguins might be too large to swallow, especially for small chicks [14]. Interestingly, on Stewart Island the poor diet was not only reflected in wide-spread chick starvation but also seemed to induce secondary diseases such as *diphtheritic stomatitis* [51], i.e. the same disease which affected chick survival on the Otago Peninsula in 2004 and triggered this study. 

Whether linear foraging patterns in yellow-eyed penguins from the Otago Peninsula are associated with a diet of reduced quality remains a matter of conjecture at this stage and more research is required to substantiate this hypothesis. However, the circumstantial evidence suggests that yellow-eyed penguins are likely exposed to cascading fisheries effects where disturbances of the benthic habitat influence the assemblages of benthic species and penguin prey, which is reflected in penguin behaviour, diet composition, and subsequent impacts on reproductive outcome. 

## Supporting Information

File S1 **Three-dimensional representations of foraging tracks performed on four consecutive days (10-13 December 2012) by a single yellow-eyed penguin (bird id 17935).** Also included is track (white) of the corresponding ROV deployment on line "C" as recorded by a GPS dive logger attached to the vehicle. See also File S2 straight-line-trip-animations.*kmz* for an animated 2D representation of the tracks. The file can be opened in most common GIS software packages but is best viewed in Google Earth (http://www.google.com/earth/).
(KMZ)

File S2 **Animations of foraging trips of two yellow-eyed penguins exhibiting straight line foraging in December 2004 (bird id 14688, line “D”) and 2012 (bird id 17935, line “C”).** The file can be opened in most common GIS software packages but is best viewed using the “View Timelines” feature in Google Earth (see https://support.google.com/earth/answer/148093?hl=en for instructions).
(KMZ)
